# Rapid fixation of non-native alleles revealed by genome-wide SNP analysis of hybrid tiger salamanders

**DOI:** 10.1186/1471-2148-9-176

**Published:** 2009-07-24

**Authors:** Benjamin M Fitzpatrick, Jarrett R Johnson, D Kevin Kump, H Bradley Shaffer, Jeramiah J Smith, S Randal Voss

**Affiliations:** 1 Ecology & Evolutionary Biology, University of Tennessee, Knoxville, TN 37996, USA; 2 Department of Evolution and Ecology and Center for Population Biology, University of California, Davis, CA 95616, USA; 3 Department of Biology, University of Kentucky, Lexington, KY 40506, USA; 4 Benaroya Research Institute at Virginia Mason & Department of Genome Sciences, University of Washington, Seattle, WA 98101, USA

## Abstract

**Background:**

Hybrid zones represent valuable opportunities to observe evolution in systems that are unusually dynamic and where the potential for the origin of novelty and rapid adaptation co-occur with the potential for dysfunction. Recently initiated hybrid zones are particularly exciting evolutionary experiments because ongoing natural selection on novel genetic combinations can be studied in ecological time. Moreover, when hybrid zones involve native and introduced species, complex genetic patterns present important challenges for conservation policy. To assess variation of admixture dynamics, we scored a large panel of markers in five wild hybrid populations formed when Barred Tiger Salamanders were introduced into the range of California Tiger Salamanders.

**Results:**

At three of 64 markers, introduced alleles have largely displaced native alleles within the hybrid populations. Another marker (*GNAT1*) showed consistent heterozygote deficits in the wild, and this marker was associated with embryonic mortality in laboratory F2's. Other deviations from equilibrium expectations were idiosyncratic among breeding ponds, consistent with highly stochastic demographic effects.

**Conclusion:**

While most markers retain native and introduced alleles in expected proportions, strong selection appears to be eliminating native alleles at a smaller set of loci. Such rapid fixation of alleles is detectable only in recently formed hybrid zones, though it might be representative of dynamics that frequently occur in nature. These results underscore the variable and mosaic nature of hybrid genomes and illustrate the potency of recombination and selection in promoting variable, and often unpredictable genetic outcomes. Introgression of a few, strongly selected introduced alleles should not necessarily affect the conservation status of California Tiger Salamanders, but suggests that genetically pure populations of this endangered species will be difficult to maintain.

## Background

Historically, most evolutionary biologists had been comfortable with a concept of "the genome" as a unitary and relatively constant feature of a species. In this view, extensive networks of biochemical and regulatory interactions were thought to result in "coadapted gene complexes" that are rather sensitive to disruption by mutation or recombination with a foreign genome [1,2]. Alternatively, in sexually reproducing populations, individual genomes can be seen as temporary confederations of alleles inherited from various ancestors across generations of independent segregation and reassortment [3,4]. While these latter processes can have the effect of homogenizing contemporary gene pools [5,6], the reshuffling that occurs with each sexual generation also allows different loci to follow different genealogical histories and respond differently to the effects of mutation, drift, selection, and gene flow [7-10]. Studies of hybridization and gene flow indicate that there is a certain amount of truth in both views. Genetic interactions often cause hybrid inviability and sterility [11-13]. However, studies of wild populations also support the idea that many alleles can pass freely between differentiated gene pools [14,15] and that recombination between divergent genomes can produce highly fit individuals [16-18]. These ideas are not new, but they are receiving a new level of attention in light of genome-scale datasets. In particular, the possibility that different processes might simultaneously hold sway over different loci such that hybrid dysfunction, hybrid vigor, and rapid introgression may all be occurring in the same set of introgressed populations can now be examined even in non-model systems.

One of the major hurdles for both laboratory and field analyses of hybridization is that some of the most important and interesting processes occur quickly, offering a narrow window of opportunity to study them. In fact, the long-standing, natural hybrid zones that form the core of most empirical research presumably represent examples of secondary contact that have *not *resulted in the alternative outcomes of reinforcement, extinction, or fusion of young lineages. Analyses of these "standard" zones have provided tremendous insights into those genes and characters that remain differentiated in the face of hybridization [19-22]. However, they are potentially a biased subset of the genes and characters that differed *prior *to secondary contact. In particular, genes that afford the greatest fitness gains for both hybridizing groups are expected to rapidly sweep through hybrid zones, leaving, at best, evidence of reduced variation as an indication of their recent dynamics [23]. Such "foreign" alleles that become fixed in a new population or species will often be viewed *post hoc *not as the result of hybridization, but simply as ancestral shared states that never diverged. However, unraveling the history of such genes is critical, because cross-taxon transfer of adaptive traits is one of the major positive consequences of introgressive hybridization [16,24,25]. Our research on a recently established hybrid zone offers the rare opportunity to observe the process of secondary contact early, while good pre-contact reference populations still exist and before such sweeps have gone to fixation in nature.

Hybridization in the wild between California Tiger Salamanders (*Ambystoma californiense*) and Barred Tiger Salamanders (*A. tigrinum mavortium *or *A. mavortium*) began in the 1940's when Barred Tiger Salamanders were imported from Texas and released near Salinas (Monterey County, CA) in a deliberate attempt to improve the local bait fishery [26]. About 20 generations of admixture have ensued in the Salinas Valley with gradual spreading of the hybrid swarm [27]. Large reference populations persist, representing the historically pure genotypes of both the native [28] and introduced taxa. This system provides the opportunity to identify genetic differences that have accumulated between groups that had been geographically isolated for several million years [29], and to observe the status of those differences after 60 years, or about 20 generations, of recombination, genetic drift, and natural selection.

We assayed alternative population genetic outcomes in the wild by testing a large set of species-specific, mapped molecular markers for deviations from Hardy-Weinberg expectations (HWE), linkage disequilibria (LD), and heterogeneity in the frequencies of introduced alleles. Our goal was to identify deviations from patterns expected under a neutral admixture process in order to evaluate the relative importance of deterministic and stochastic processes in shaping contemporary genetic variation. We also scored this same set of markers in captive-bred F1 and F2 hybrids to confirm the utility of the markers for characterizing hybrid genotypes and to identify markers potentially affected by selection before hatching.

## Results

### F2 Hatchlings

We generated two F2 families and genotyped a sample of hatchlings for all markers. We also genotyped four controls, including both pure parentals, an F1, and a blank. Only one marker (*GNAT1*) exhibited significant deviations from Mendelian expectations in both F2 samples (Table 1). Because embryonic mortality was high (139 of 506 in the first cross and 133 of 484 in the second cross; mean survival = 0.742), differential embryonic survival might account for the deviation from Mendelian ratios. Alternatively, prezygotic factors such as meiotic drive or assortative fertilization might cause deviation from Mendelian ratios. F1 hybrids showed an even lower embryonic survival rate of 44.8% (260 failed to hatch of 471 eggs from a single cross). These F1 and F2 embryonic survival and genotype frequency results are consistent with reduced *GNAT1 *heterozygote survival. If these heterozygotes suffer about 50% mortality as embryos but homozygotes have close to zero mortality, this would lead to about 50% mortality of F1's (all of which are heterozygotes) and 25% mortality of F2's (which are half heterozygotes) and result in even (1:1:1) genotype frequencies in the surviving F2's. The genotype frequencies in Table 1 are not significantly different from 1:1:1 (χ^2 ^= 2.17, df = 2, *p *= 0.338).

**Table 1:** Genotype frequencies of the *GNAT1* marker in  F2 hatchlings

	CC	CT	TT	χ^2^	*P *(df = 2)
Family 1	35	29	26	13.18	0.00138
Family 2	32	30	25	9.51	0.00863
Combined	67	59	51	22.56	0.00001

The native allele is represented by "C" and the introduced allele by "T". The χ^2 ^test compares the observed counts to Mendelian expectations (1:2:1). Family 1 and Family 2 are not significantly different (χ^2 ^= 0.12, df = 2, *P *= 0.9417).

### Allele Frequencies in the Wild

**Figure 1:**
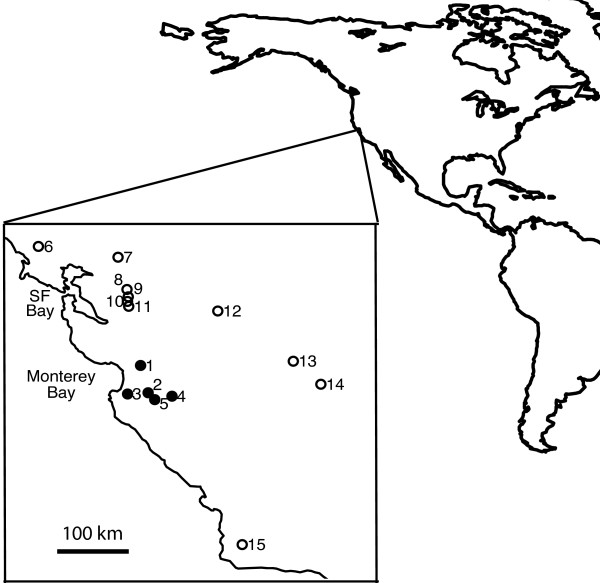
**Study sites**. Approximate locations of study sites (black filled circles) and native "control" sites (open circles) in California, USA. Dashed lines illustrate regions previously known to have some level of introgression [27]. Sites are: 1 – Bluestone, 2 – Sycamore, 3 – Toro, 4 – Melindy, 5 – Pond H, 6 – Ludwig, 7 – Olcott, 8 – Diablo, 9 – Vasco, 10 – Frick, 11 – Tesla, 12 – Hickman, 13 – Urrutia, 14 – Alta, and 15 – Black.

We sampled larvae from five wild populations (Figure 1). These sites are within the region of known historical introductions and all are livestock ponds that dry up over the summer [27]. Sampling of larvae guarantees a single breeding-season cohort that includes no first generation immigrants. However, as discussed below, the mating system of tiger salamanders (where few breeding pairs might each produce hundreds of offspring) can generate deviations from Hardy-Weinberg and linkage equilibria.

**Figure 2:**
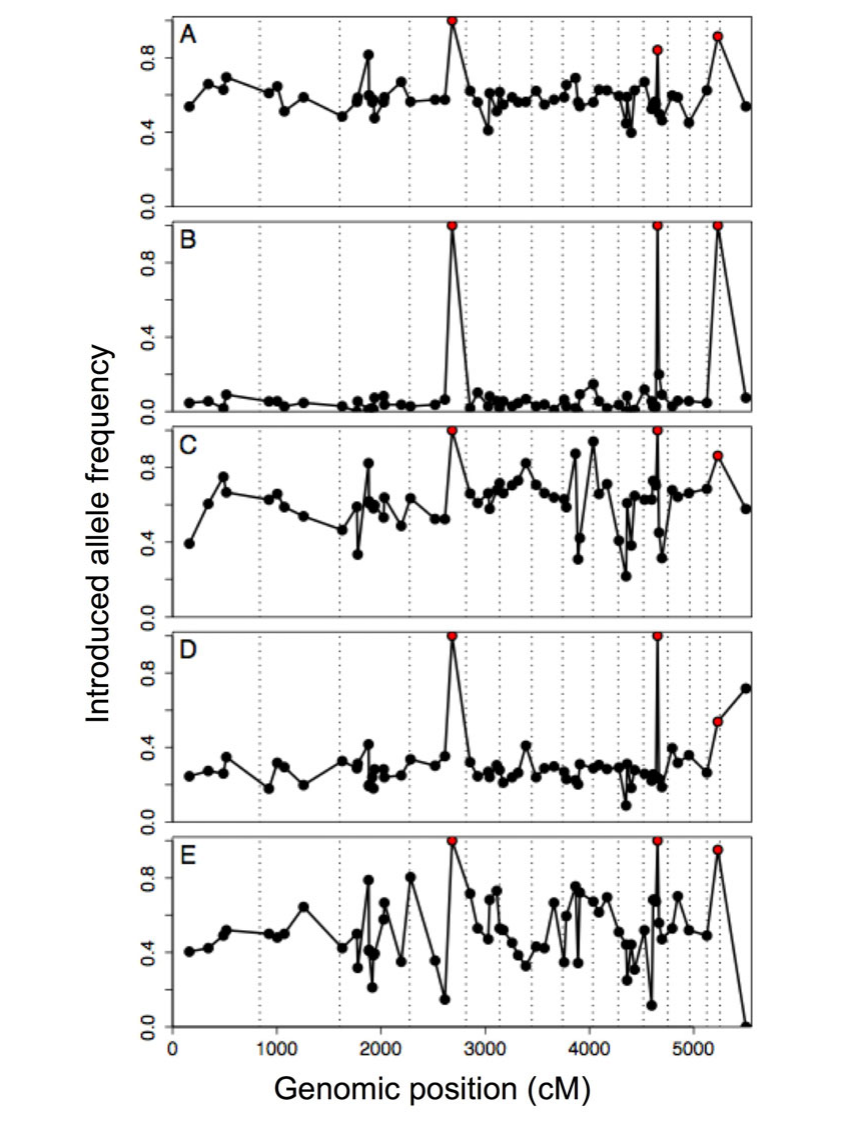
**Allele frequencies**. Frequencies of introduced alleles by position in the *Ambystoma *linkage map [36] and by study pond: (A) Bluestone, (B) Melindy, (C) Pond H, (D) Sycamore, (E) Toro. Red points are markers (from left to right) E23C06, E06E11, and E12C11. Vertical dashed lines separate linkage groups, which are plotted end to end from largest to smallest [36]. The last marker is the mtDNA marker, placed arbitrarily at the end of the linkage map.

The five ponds vary in their level of genetic invasion, from Melindy (average introduced allele frequency = 0.095) to Pond H (0.621), and most of the 64 markers frequencies fell within their predicted population variance (Figure 2, Table 2). In contrast, markers E06E11, E12C11, and E23C06 had consistently high frequencies of introduced alleles, with E23C06 fixed for introduced alleles in all samples (Figure 2, Table S1 [see Additional file 1]). Unconditionally beneficial alleles are expected to move rapidly toward fixation in hybrid populations. As a result, the distribution of allele frequencies would be distorted relative to its expectation if genetic drift alone were responsible for allele frequency changes subsequent to admixture [30]. Long's [30] test for heterogeneity of admixture rejects the null hypothesis of neutral admixture when the tails of the empirical distribution of admixture estimates are thicker than expected from the variance. Applying Long's heterogeneity test to our data, we reject neutral admixture in all five study ponds (Table 2). Furthermore, the same markers are consistently responsible for the deviations in all five study ponds (Table S1 [see Additional file 1]). Dropping the three markers with the most extreme allele frequencies and recalculating Long's test yields a non-significant result for all ponds except Toro, which required additionally dropping the mtDNA marker to achieve adequate goodness-of-fit (*P *> 0.05) to the neutral admixture model (Table 2).

**Table 2:** Long's test for heterogeneity of admixture rates

Pond	*n*	*M*	*F*_LS_	X^2 ^(63 df)	X^2 ^(60 df)	X^2 ^(59 df)
Bluestone	41	0.591	0.041	588.4 ***	26.1	
Melindy	55	0.095	0.486	1120.5 ***	31.1	
Pond H	51	0.621	0.094	412.5 ***	61.4	
Sycamore	56	0.306	0.108	1786.7 ***	23.6	
Toro	52	0.518	0.148	1058.6 ***	414.8 ***	53.3

Sample size (*n*), mean admixture proportion (*M *= introduced allele frequency across all markers), standardized variance of the introduced allele frequencies among markers within samples (*F*_*LS *_– analogous to *F*_*ST *_among subpopulations within a metapopulation [30]), and Long's test statistic for goodness-of-fit to the null hypothesis of neutral admixture (accounting for variation owing to drift). The test with 63 df includes all markers, the test with 60 df eliminated the three most introgressed markers (E06E11, E12C11, and E23C06), and the test with 59 df also eliminated mtDNA (Toro only). *** *P *<< 0.0001

Because the validity of Long's test might be compromised when the variance among markers is large [30] or when typical population genetic assumptions are not met, we performed a series of simulations to evaluate whether our results were likely to occur by genetic drift alone (Figure S1 [see Additional file 2] and Methods). Simulations never recovered patterns resembling our observations (Figs. S2–S4 [see Additional file 2]), reinforcing our inference that the extreme allele frequencies observed for markers E06E11, E12C11, and E23C06 cannot be explained by genetic drift.

### Deviations from HWE

**Figure 3:**
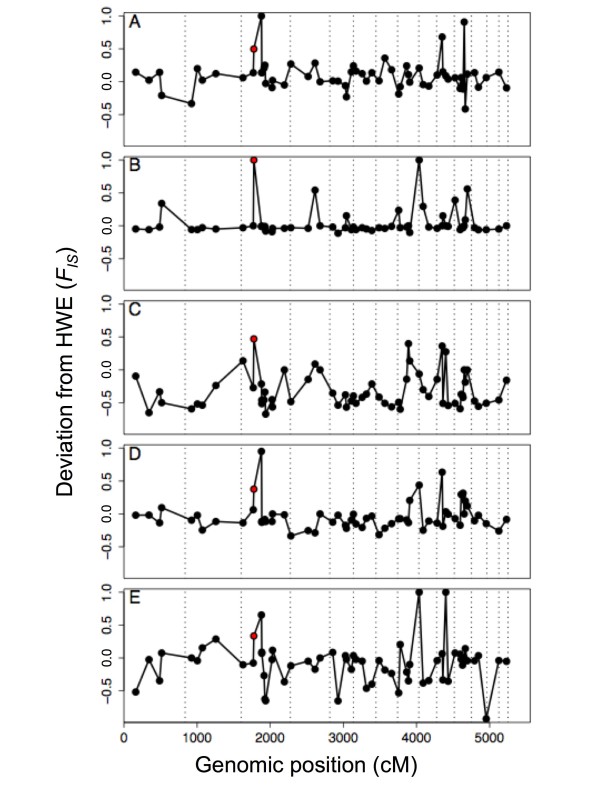
**Deviations from Hardy-Weinberg expectations**. Deviation from Hardy-Weinberg expectations (HWE) by position in the *Ambystoma *linkage map [36] and by study pond: (A) Bluestone, (B) Melindy, (C) Pond H, (D) Sycamore, (E) Toro. Positive *F*_IS _indicates heterozygote deficit and negative *F*_IS _indicates heterozygote excess. The red point is *GNAT1*. Vertical dashed lines separate linkage groups, which are plotted end to end from largest to smallest [36].

Deviations from HWE were common, but generally inconsistent among ponds (Figs. 3 and S5 [see Additional file 2]). Most markers showed heterozygote excess in Pond H (Figure 3), but the other four ponds did not generally deviate from zero. Few strong deviations were apparent in Melindy because most markers were nearly fixed for native alleles in that pond (Figs. 2 and 3); this is a consequence of its unique introduction history (see Methods). Beta-uniform mixture analysis (BUM; [31]) of among-pond heterogeneity tests indicates that the null hypothesis of equal *F*_IS _among ponds is likely to be correct for only 15 markers (23%). Overall, deviations from HWE were not strongly correlated among ponds (Figure S6 [see Additional file 2], Table S2 [see Additional file 3]) and we suggest that the wide variance of *F*_IS _is largely a consequence of small effective breeding populations combined with large clutch sizes (see Discussion).

Only *GNAT1*, the marker with a heterozygote deficit in F2 hatchlings (Table 1) showed heterozygote deficiencies in all wild population samples (Figure 3, Table S1 [see Additional file 1]). The same pattern was noted previously for a different set of wild populations [17,27,32].

### Linkage Disequilibria

Selection against recombinant genotypes with incompatible alleles should generate non-random associations (linkage disequilibria) between conspecific alleles of different markers (e.g., [13]). Linkage disequilibria (LD) are also created by admixture and gene flow between populations with divergent allele frequencies [33]. Admixture LD decays each generation according to the recombination rate between loci. Therefore, LD is affected by the history of admixture and gene flow among local breeding populations in addition to ongoing selective processes and drift [34,35]. Thus, exceptional pairs of loci must be identified with respect to the empirical distribution of pairwise linkage disequilibria and compelling examples should be consistent among breeding ponds.

**Figure 4:**
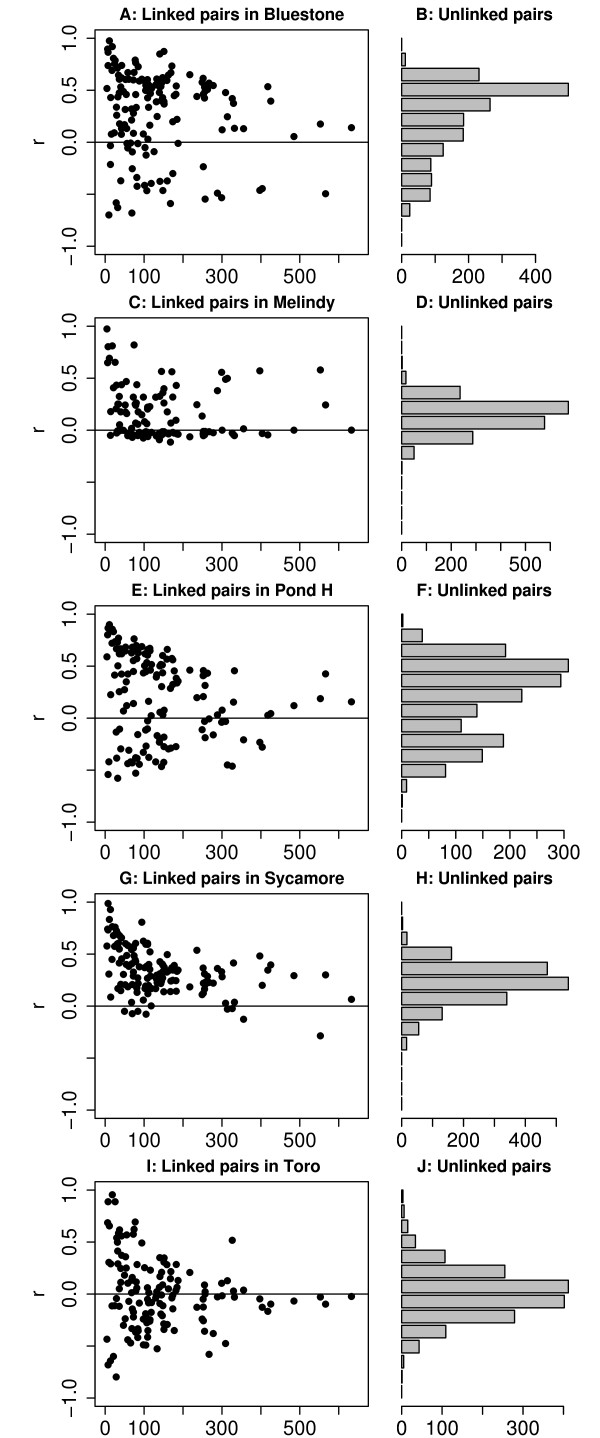
**Linkage disequilibria**. Linkage disequilibria between pairs of markers by study pond. "Linked" pairs (those on the same linkage group) are plotted against their recombinational distance. Unlinked pairs (those on different linkage groups) are plotted in the histograms.

In our data set, the distribution of LD between physically unlinked markers (those on different linkage groups according to [36]) shows a wide variance in all ponds and positive bias in four of five ponds (Figure 4). LD in these wild hybrid populations is only weakly related to map distance within linkage groups, but only 32 pairs of markers had expected recombination rates of less than 1/2 (under 50 cM in Figure 4). For unlinked markers, there was no general correlation of LD coefficients among ponds (Figure S7 [see Additional file 2], Table S3 [see Additional file 3]) and no specific pairs of markers stood out as having consistently high or low LD. For example, no pair of markers was consistently outside of the 95% quantile envelope (Figure S7 [see Additional file 2]).

## Discussion

Hybridization between the native California Tiger Salamander and the introduced Barred Tiger Salamander over the last 60 years has resulted in a complex hybrid swarm composed of many local, semi-independent breeding populations. The genetic dynamics of such admixed populations is an important area of study in both evolutionary and conservation biology and might be different from dynamics in long-standing natural hybrid zones in important ways. In particular, neutral admixture, genetic swamping, reinforcement, stable hybrid zones, and hybrid vigor have often been characterized as mutually exclusive, population-level outcomes of hybridization. However, the population genetic processes underlying those outcomes can co-occur, each influencing different genomic regions to different degrees. Recombination can facilitate diverse responses to selection across different genomic regions. We have found evidence that different loci have undergone different population genetic processes within the same populations, illustrating that the simple typology of "native", "introduced", and "hybrid" is not an adequate framework for addressing the evolutionary dynamics and conservation implications of this biological invasion (and potentially many others).

We detected few deviations from Mendelian genotype proportions in captive-reared F2 hatchlings. Although the small number of F2 families studied constrains our ability to make broad inferences, the only significant pattern – heterozygote deficit at the *GNAT1 *marker – has already been observed in many samples from wild populations [17,27,32], and we take the general lack of deviations from Mendelian proportions at other markers as an encouraging sign that our markers are valid ancestry informative markers with Mendelian inheritance. In contrast, many deviations from neutral, equilibrium expectations were evident in the data from wild breeding populations. These patterns suggest that natural selection and complex demographics strongly influence genetic variation in the wild.

### Fixation of alleles

Our most striking result is that native alleles have been completely replaced by introduced alleles at one marker in our five study sites, and that two other markers are not far behind (Fig 2). Although no markers showed the opposite pattern, the numbers are too small to support an inference that loci with favorable introduced alleles are more numerous than loci with favorable native alleles.

Long's [30] heterogeneity test indicated that the three markers with the most extreme allele frequencies represent significant deviations from the distribution of allele frequencies expected under neutral admixture (Table 2). An important alternative to consider is that the extreme markers were not actually diagnostic with respect to native or non-native ancestry. Our California Tiger Salamander controls were chosen from throughout the native range, including all major phylogeographic groups [28]. If the patterns that we attribute to introgression are instead due to natural variation within the native California Tiger Salamander, it would have to be the case that native alleles identical in state to introduced alleles were common only in the geographic areas where the introductions have been documented to occur [27]. Putative introduced alleles of E06E11 were not found in any native control sites. For E12C11, the introduced SNP was found at Diablo and Tesla and for E23C06, introduced alleles were detected at Tesla. Both of these sites are very close to the previously hypothesized northern range edge of the introduced hybrid swarm (Figure 1). The fact that these introduced alleles were found only in populations in close proximity to the edge of the hybrid swarm, and nowhere else in the native range of the species, strongly suggests that these results are best explained by introgression. We suspect that highly advantageous introduced alleles are linked to these markers and they are in the process of spreading throughout the range of the California Tiger Salamander. While only a minor genetic change (~5% of markers), the phenotypic consequences of these alleles are unknown.

### Heterogeneity among breeding ponds

Deviations from Hardy-Weinberg and linkage equilibrium were common, but very idiosyncratic (Figs. 3 and S5 [see Additional file 2]), as noted previously in this system [32]. The breeding ecology of tiger salamanders may accentuate random deviations from Hardy-Weinberg and linkage equilibrium because random mating is not equivalent to random union of gametes when a single mating produces more than one offspring. High fecundity of single matings violates a key assumption of the Hardy-Weinberg principle, that offspring are formed independently by random union of gametes [33,37]. In tiger salamanders (and many other organisms, including pond-breeding amphibians) a single mating can produce hundreds of full siblings [38,39]. This family structure is a potential source of non-independence of larvae within ponds, even if families are formed by random mating and there is equiprobable survival of all offspring. Just as mixture of two or more population samples will tend to cause the appearance of heterozygote deficits (the Wahlund effect, [33]), a family-level Wahlund effect is generated when larval populations are mixtures of a few large sibships.

Our simulations demonstrate that this breeding system can increase the variance of *F*_IS_, and therefore the probability of rejecting HWE. For example, we simulated breeding by 10 random pairs from a potential pool of parents with two equally common alleles in HWE (see  for code). Each pair generated 500 offspring by random union of parental gametes (i.e., following Mendel's law of segregation). We then drew a simple random sample of 50 individuals from the 5000 offspring and tested the sample for deviation from HWE. Both the traditional χ^2 ^and Fisher's exact tests rejected the null hypothesis in about 200 of 1000 replicates (Figure S8 [see Additional file 2]). The distribution of *F*_IS _in this simulation is clearly wider than the distribution generated when we simulated true random union of gametes with the same sample size and population size (5000 random pairings, each yielding a single offspring). The result is not an artifact of sampling (censusing all 5000 offspring shows that most replicates violate HWE), but is sensitive to the breeding population size (50 random pairs, giving 500 offspring each, resulted in genotype frequencies indistinguishable from HWE 94% of the time). Thus, significant heterogeneity of *F*_IS _might be evidence of small effective population sizes relative to the larval census populations.

Clutch sizes in tiger salamanders (both native and introduced) often exceed 1000 eggs [38-40] and breeding females numbered fewer than 10 in most ponds in most years for the native California Tiger Salamanders observed by Trenham et al. [41]. Thus, populations of tiger salamander larvae might often consist of many members of a few large families rather than many genetically-independent offspring. Under such demographic conditions, random deviations from HWE are expected to be exaggerated. This result emphasizes the importance of assaying several breeding ponds and/or a single pond for multiple breeding seasons to test for consistency of deviations from equilibrium expectations.

### Candidate markers and modes of selection

We identified markers *GNAT1*, E06E11, E12C11, and E23C06 as being potentially linked to genetic loci under selection in hybrid tiger salamander populations. These markers are not physically linked [36], implying that each indicates the existence of a separate chromosomal region strongly affecting hybrid fitness. It is important to note that our markers are sparsely distributed over the *Ambystoma *linkage map and there is no reason to think that the markers showing evidence of selection are actually the targets of selection. Finer scale mapping will be necessary to identify specific DNA sequences affecting fitness variation in these populations; however, more time might be necessary for recombination to break up fine scale LD.

The marker *GNAT1 *has been noted before for showing heterozygote deficiency in wild populations [17,27,32]. This study includes many times more markers than previous work, and *GNAT1 *remains exceptional. The F2 data indicate that the heterozygote deficit arises prior to hatching, most likely as a consequence of embryonic mortality. We propose that *GNAT1 *is linked to one or more genes or a chromosome rearrangement causing massive mortality in hybrid embryos. Although the partial postzygotic isolation caused by embryonic mortality is not adequate to prevent extensive genetic mixing, further study of this genomic region might yield valuable insights into the nature of the genetic differences causing hybrid dysfunction. For example, if embryonic mortality is caused by heterozygote disadvantage at a single gene, this would be the first counter-example to the proposition that most hybrid mortality and sterility is caused by Dobzhansky-Muller interactions between genes [12,42-44].

While it is tempting to attribute population genetic patterns to specific modes of natural selection, there is not a one-to-one relationship between fitness and genotype frequency. For example, heterozygote deficits might reflect underdominance at single loci, but can also be caused by epistatic interactions between loci (Figure S9 [see Additional file 2]) and prezygotic barriers to heterozygote formation. Rapid fixation of an allele in a local population might be caused by simple directional selection, but can also be driven by underdominance or epistasis (Figure S9 [see Additional file 2]). As underdominance and epistasis tend to have frequency-dependent effects, a broader geographic analysis of the tiger salamander hybrid zone might distinguish patterns owing to universally advantageous fitness effects from effects that depend on the initial frequencies of alleles or the individual or population genetic background. An attempt at such an analysis is currently underway by our group.

### Conservation implications

The California Tiger Salamander is listed as threatened under the US Endangered Species Act, so both ecological and taxonomic consequences of hybridization and introgression are clear and present challenges for conservation management [17,26,27,45-47]. Ecological consequences should be paramount when assessing the impact of a biological invasion [48], particularly for species like tiger salamanders which are top predators in sensitive vernal pool ecosystems. A recent experimental study indicates that tiger salamanders with high levels of introduced ancestry have much greater rates of predation on other native amphibians compared to native California Tiger Salamanders [49]. A critical question is whether such impacts are likely to be restricted to highly admixed populations or whether largely native populations like Melindy, which contain a few, rapidly spreading introduced alleles, are also expressing undesirable ecological traits. Minor genetic differences can underlie major life history differences in the tiger salamander complex [50,51]. It might be that traits subject to strong positive selection are disproportionately likely to have negative impacts on other community members.

In addition, two strictly genetic questions arise when native and introduced taxa hybridize. First, should any genetic mixing, at any frequency, be considered a biological threat to the native taxon? When native genotypes and alleles are replaced by mixed-ancestry genotypes, there is both an overall loss of biological diversity and a local gain in genetic diversity; the former represents the amalgamation of previously distinct lineages of tiger salamander, while the latter occurs locally in California. Some authors have characterized such genetic change as partial or complete extinction [24,52]. However, such "genetic extinction" is not the same as demographic extinction (where all individuals die without leaving offspring); there are still naturally reproducing populations that have many of the biological attributes and genes that characterize the native species. At least in a demographic sense, introgression of non-native alleles cannot be objectively characterized as against the best interests of wild salamanders. Second, what is the legal status of mixed genotypes when one parental lineage is protected? Current policy is to decide on a case-by-case basis [45] and the USFWS approach to hybrid tiger salamanders has been loosely characterized as protection based on "similarities" rather than ancestry per se [53]. If that similarity is purely genetic, then introgression of a few introduced alleles should not change the legal status of California Tiger Salamander populations. If the key similarity is morphological, ecological, or behavioral, then it implies that those characters, perhaps in light of their genetic underpinnings, should be the cornerstones of management decisions.

## Conclusion

One of the most fundamental problems in the study of hybrid zones is to understand the effects of heterogeneity among different portions of the genome on the dynamics of secondary contact. After a few generations of admixture, there are thousands of recombinant hybrid genotypes, each with a potentially different phenotype and fitness. Some long-standing hybrid zones exhibit remarkable concordance across traits and loci [54,55], while others show complex variation among parts of the genome [14,56-58]. Our results indicate that about 5% of markers are rapidly approaching fixation for a non-native genotype in the center of the tiger salamander hybrid zone in California. This observation might have been obscured entirely after a few more generations of selection and dispersal; once fixed throughout the range of the California Tiger Salamander, an introduced allele would not be recognized as such without a historical record of genetic change. At least until the phenotypic and ecological consequences of such changes are clarified, we advocate the view that small amounts of introgression represent small evolutionary changes that should not change the fundamental management of endangered species. Though such changes might be undesirable, they do not constitute true extinction, nor should they disqualify introgressed populations from protection [59].

## Methods

### Marker Development

To identify single nucleotide differences between California Tiger Salamanders and Barred Tiger Salamanders, we sequenced markers derived from the EST library described by Smith et al. [36]. Initial EST sequences were chosen to be approximately evenly spaced at roughly 50 cM intervals on the *Ambystoma *linkage map [36]. Sequences from four individuals each of *A. californiense *and *A. tigrinum mavortium *were compared to existing sequences for the Axolotl (*Ambystoma mexicanum*) and Eastern Tiger Salamander (*A. t. tigrinum*) available at . Because these latter two taxa are more closely related to Barred Tiger Salamanders than California Tiger Salamanders [29], we reasoned that nucleotides differentiating California Tiger Salamanders from all three of the others were most likely to be fixed differences that evolved before the divergence of *A. mexicanum, A. t. tigrinum *and *A. t. mavortium*. These candidate nucleotides were then scored in 16 pure California Tiger Salamanders from throughout their native range (Figure 1) and 8 pure Barred Tiger Salamanders from a pure introduced population in Lake County, CA (Table S4 [see Additional file 3]). This introduced population is known to be from the same introduction source as those in this study, but exists outside of the native California Tiger Salamander range. Using binomial 95% confidence intervals [60], we can be confident that if a marker was diagnostic in this test panel, the natural frequency of "introduced" alleles in California was less than 11% and the natural frequency of "native" alleles in the introduced population was less than 20%. The only marker known to be non-diagnostic was *GNAT1*, for which Barred Tiger Salamanders have a very low frequency (0.02–0.04) of the "California" allele [32]. We still treat *GNAT1 *as effectively diagnostic, as this level of polymorphism adds only a small amount of noise to our analyses.

For this work, we sought markers that were reliably amplified and easy to score for the three diallelic genotypes using the FP-TDI method [61]. After screening out markers that were not diagnostic or could not be scored reliably, 64 markers were used: 54 new markers from the *Ambystoma *genetic map [36], eight nuclear markers and one mitochondrial marker used previously [32], and the nuclear marker *contig325 *which is closely linked to a major-effect QTL implicated in the evolution of paedomorphosis in *A. mexicanum *[51]. The EST-based markers and *contig325 *were scored at the University of Kentucky and the nine previously used markers were scored at U. C. Davis. Sequences, PCR primers, and details of the SNP assay for each marker are available on the Ambystoma Research Network website .

### F2 Hybrids

To verify the reliability of marker scoring and Mendelian inheritance of alleles, all nuclear markers were scored in a family of F2 hybrids using a separate molecular facility at the University of Tennessee. We generated F2 hybrids by allowing pairs of F1 hybrids to mate in outdoor tanks at U. C. Davis. We housed fertilized eggs in clean water until hatching, euthanized hatchlings in MS-222, and then extracted DNA from each hatchling using Promega's SV 96 DNA purification kit . An initial set of 92 F2 hatchlings was scored for all 63 nuclear markers (all were known to have California Tiger Salamander mtDNA). Pure, F1, and negative controls were included in each 96-well plate. Genotypes were called using the macro for Microsoft Excel provided by Perkin-Elmer . Data for each marker were tested against the null hypothesis of Mendelian ratios (1:2:1) using a standard χ^2 ^test with 2 degrees of freedom [62,63]. Markers with χ^2 ^exceeding the Bonferonni adjusted critical value of 14.29 were scored in a second set of 92 F2 hatchlings from a different pair of F1 parents.

### Wild Hybrids

We sampled five wild breeding sites for population genetic analysis (Figure 1, Table 2). Larvae were captured haphazardly using a 3 m seine and 1 cm of tail fin tissue was collected for DNA analysis (preserved in ethanol). Larvae were then immediately released. DNA was extracted using the standard phenol-chloroform protocol [64].

The sites were all seasonal cattle ponds. Based on prior analysis of a smaller set of markers [27], we chose these sites to cover a range of allele frequencies. Melindy Pond is the most exceptional in having very low frequencies of introduced alleles. This pond was pure native until the early 1970's when a bait salesman deliberately stocked it with hybrid larvae collected from the Salinas area (Mr. Don Green, pers. comm.). The other four ponds are within the region where bait salesmen intentionally released larvae from Texas and Colorado in the 1940's (Mr. Don Green, pers. comm.).

### Statistical Analyses

To address the question of whether directional selection has affected the frequency of introduced alleles at specific markers, we used Long's test statistic for heterogeneity of admixture among markers [30]. This test incorporates both sampling variance and "evolutionary variance" owing to genetic drift into its null hypothesis. Genetic drift alone will cause increasing variance in allele frequencies after admixture until eventually all loci are fixed for one or the other of the parental alleles. However, directional selection will accelerate fixation of favored alleles and this will distort the distribution of allele frequencies. Long's test statistic is expected to be χ^2 ^distributed when drift and sampling alone are responsible for the variance among markers. This null hypothesis can be rejected when some marker frequencies are outliers relative to the observed distribution of allele frequencies within a population. A recently developed method with the same main goal does not account for genetic drift after admixture and is prone to Type I error for data sets such as ours, where drift is likely to have influenced the variance among markers [65].

Replication among ponds is necessary to test for a deterministic cause for extreme allele frequencies. To quantify the contribution of each marker to each population test statistic, we recorded the χ^2 ^residual (*R*_*i*_) for each marker *i*, which in this case is



where *M*_*i *_is the estimated frequency of introduced alleles at marker *i*, *M *is the average across all markers, and *V*(*M*_*i*_) is the variance of the estimator, taking account of both sampling and drift following Long's [30] equation 18. We then compared marker residuals among ponds by converting each to a signed effect size (φ_*i *_= *R*_*i*_/√*n*_*i*_, where *n*_*i *_is the sample size for the ith marker).

If a marker consistently contributed to deviation from the neutral expectation, then we may infer that its allele frequency has been influenced by natural selection. We identified markers with disproportionate effects by inspection of effect sizes (Table S1, [see Additional file 1]) and by dropping markers from the analysis and observing the behavior of Long's test on the reduced data set.

In addition, Long's test might not be valid when the variance among markers is large [30] and the structure of larval salamander populations (potentially a few large sibships) might accentuate the influence of genetic drift. Therefore, we examined the distributions of Long's *X*^2^, the variance among markers (*F*_*LS*_), and the number of fixed markers in simulations of admixture and drift in populations with overlapping generations, small numbers of breeders, and large clutch sizes (meant to capture the ways tiger salamanders deviate from typical population genetic assumptions). We used admixture proportions estimated from our study sites (Table 1) as initial conditions for five sets of simulations. Simulated sample sizes also followed our study sites (Table 1). For each of these five sets, we simulated, for 80 years, populations composed of 2, 4, 8, 16, and 32 breeding pairs drawn from the previous three generations. Every simulated generation, we used a simulated sample to calculate the number of fixed markers (out of 64), *F*_LS_, and *X*^2^. Simulations were repeated 1,000 times for each of the 25 combinations of initial conditions and breeding population sizes. See Figure S1 for more detail. Code, written in R, is available at .

Deviations from HWE for each nuclear marker in each pond were quantified as *F*_IS _and tested via Fisher's exact test [66]. With 63 nuclear markers, we expect 3 to 4 markers per pond to have *P*-values less than 0.05 by chance alone. To avoid spurious inference, we adopted two multiple-test strategies. First, within ponds, we tested the distribution of *P*-values against the uniform (0,1) distribution expected when the null hypothesis is true for all tests, and used the BUM method [31] to estimate the fraction of markers for which the null hypothesis of HWE is likely to be true. This method finds a mixture of a uniform (0,1) and a beta distribution that is most consistent with the empirical distribution of *P*-values. Second, we used the heterogeneity χ^2 ^test to evaluate the consistency of each marker's deviation among ponds. Heterogeneity *P*-values were again tested against the expected uniform (0,1) distribution and the BUM method applied to estimate the fraction of markers with consistent (homogeneous) deviations from HWE.

We quantified linkage disequilibrium as *r*, the correlation for each pair of markers in each pond [66]. Analysis of this measure does not require the assumption of single-locus HWE and is relatively insensitive to variation in allele frequencies [67,68]. Genome-wide linkage disequilibria are generated by admixture and maintained by ongoing gene flow between populations with divergent allele frequencies [33]. Because the tiger salamander hybrid swarm includes a mixture of breeding ponds with high and low frequencies of introduced alleles [27,32], gene flow among local ponds will tend to maintain positive disequilibria between conspecific alleles. To evaluate overall consistency of LD among ponds, we used partial Mantel tests [69] to evaluate the correlation between the matrices of LD for each pair of ponds with recombinational distance between markers as the covariate. To address the question of whether certain pairs of markers consistently show extreme levels of LD, we asked whether any pair of unlinked markers had an extreme LD coefficient (*r*) in all ponds. Extreme LD coefficients were defined as the most extreme 5% in each pond.

## Authors' contributions

BMF helped design the study and collect data, analyzed the data, and drafted the manuscript. JJS and DKK helped develop EST markers and collected and processed the marker data. JRJ helped with data collection, data analysis, and manuscript preparation. HBS and SRV helped design the study, collect data, and edit the manuscript. All authors read and approved the final manuscript.

## Authors' information

Co-authors listed alphabetically.

## Supplementary Material

Additional file 1 **Supplementary Table S1**. Spreadsheet of summary statistics by pond and by marker. Click here for file

Additional file 2 **Supplementary Figures**. Nine figures illustrating simulations, results, and relationships between selection and genotype frequencies. Click here for file

Additional file 3 **Supplementary Tables S2, S3, and S4**. Tables showing cross-pond correlation of deviations from Hardy-Weinberg and linkage disequilibria. Click here for file
