# Supplementary material for: Rapid fixation of non-native alleles revealed by genome-wide SNP analysis of hybrid tiger salamanders
Source: BMC Evol Biol. 2009 Jul 24;9:176. doi: 10.1186/1471-2148-9-176 (PMC2724412; doi:10.1186/1471-2148-9-176)
Supplement: Additional file 2 — Supplementary Figures. Nine figures illustrating simulations, results, and relationships between selection and genotype frequencies. [file 1471-2148-9-176-S2.doc]

Supplementary Figures for

Fitzpatrick et al. "Rapid fixation of introduced alleles revealed by genome-wide SNP analysis of hybrid tiger salamanders"


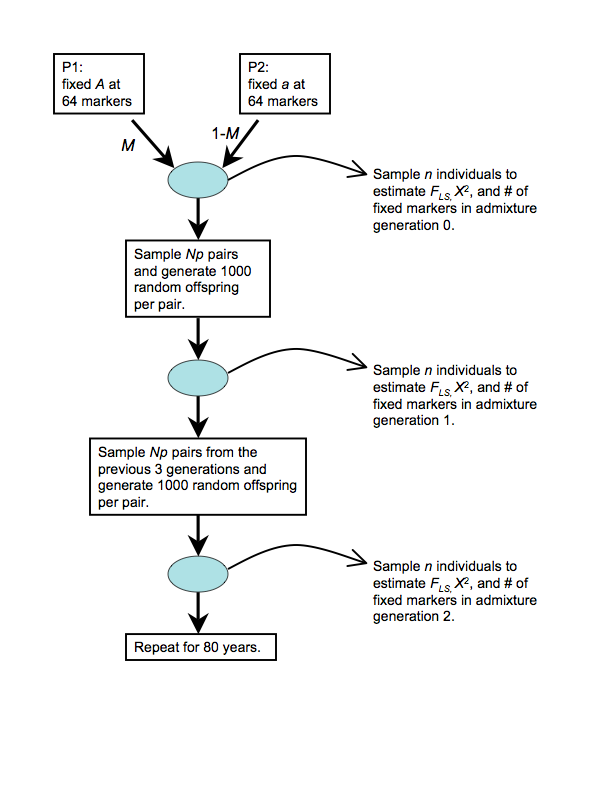


Figure S1. Schematic illustration of the admixture model used to evaluate Long's test. A single episode of admixture is followed by 80 years of genetic drift (no mutation, selection, or gene flow) with a simple model of overlapping generations (breeders are equally likely to come from the last three generations of offspring, except for the first three generations in which breeders are equally likely to come from the previous one or two generations). The admixture proportion (*M*) and sample size (*n*) were obtained from empirical estimates, generating five sets of initial conditions corresponding to our five study ponds. The number of breeding pairs (*Np*) was 2, 4, 8, 16, or 32 for a total of 25 combinations of initial conditions and *Np*. The model represents an extreme case of drift in that gene flow among populations with similar *M* would tend to dampen the effects of drift.


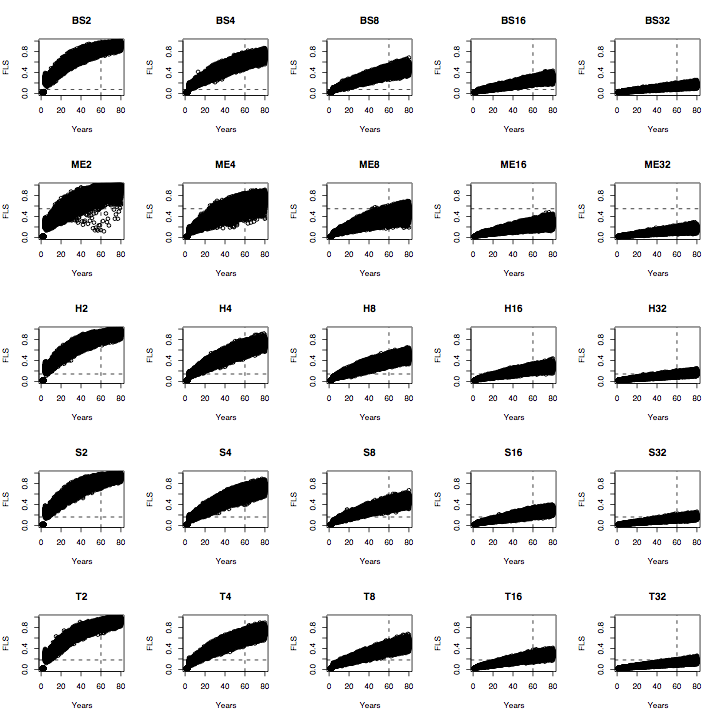


Figure S2. Accumulation of variance among markers (*FLS*) over time since admixture. Each panel represents a set of 1000 simulations of genetic drift with initial conditions based on our study sites. Rows correspond to different admixture proportions and sample sizes while columns correspond to different numbers of breeding pairs. Initial conditions are represented by capital letters: BS – Bluestone, ME – Melindy, H – Pond H, S – Sycamore, T – Toro. Breeding population sizes are represented by numbers (2, 4, 8, 16, 32 breeding pairs). For example, ME4 is simulated admixture proportion based on the average allele frequency in Melindy, sample sizes of 55 larvae examined each generation, and 4 mating pairs each generation. Dashed horizontal lines illustrate the observed value of *FLS* in the corresponding pond. The vertical line at 60 years represents the maximum time since admixture for California tiger salamander populations.


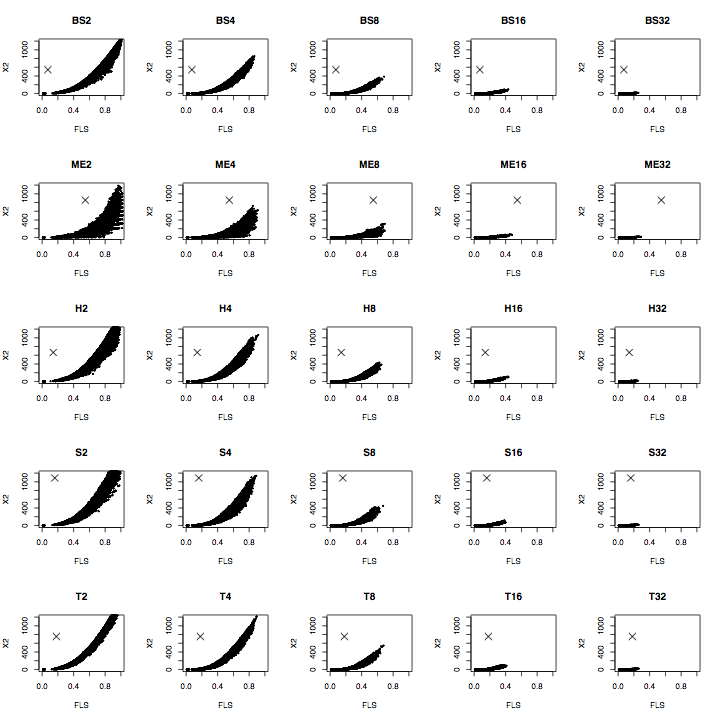


Figure S3. Joint distributions of Long’s *X*2 and *FLS* across all samples from the simulations illustrated in Fig. S1. 80,000 points for each initial condition and breeding population size include samples from each of 80 years from each of 1000 replicates. The X’s illustrate the observed values for each pond.


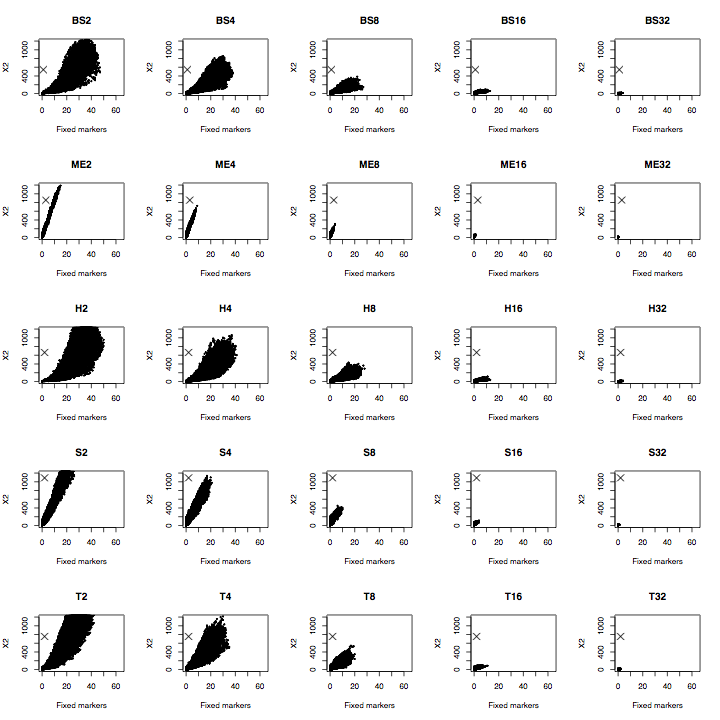


Figure S4. Joint distributions of Long’s *X*2 and the number of markers fixed for introduced alleles across all samples from the simulations illustrated in Fig. S1. 80,000 points for each initial condition and breeding population size include samples from each of 80 years from each of 1000 replicates. The X’s illustrate the observed values for each pond.


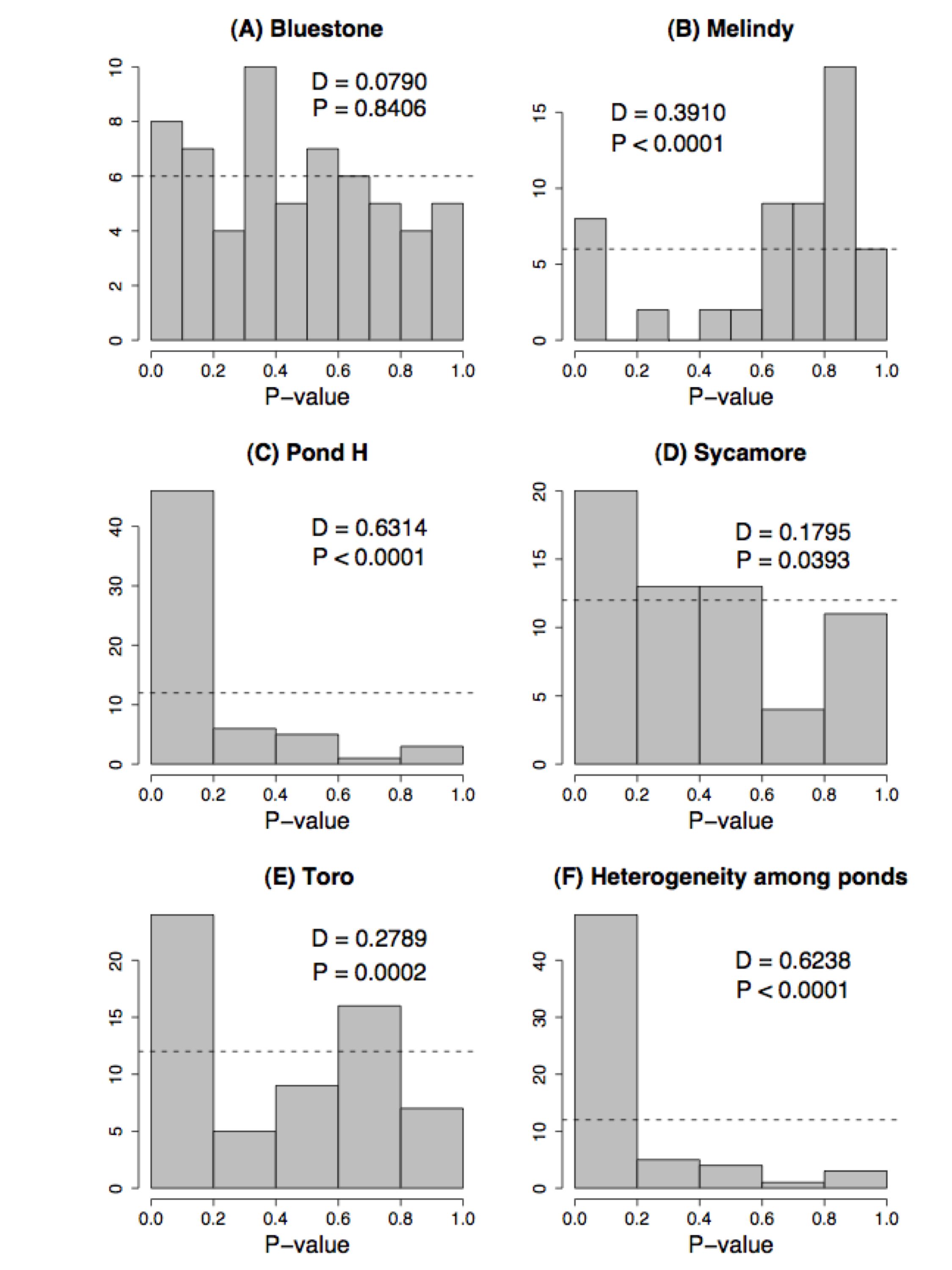


Figure S5 - Distributions (across markers) of Fisher’s exact *P*-value for goodness-of-fit to Hardy-Weinberg expectations (HWE) by study pond (A-E) and the distribution of *P*-values for among-pond heterogeniety in the deviation from HWE (F). The uniform expectation is illustrated by the horizontal dashed line in each panel. Within each panel, D and P are the kolmogorov-smirnov test statistic and *P*-value for goodness-of-fit of *P*-values to the uniform distribution. Most ponds have a large number of statistically significant deviations from HWE (A-E) but the deviation from HWE varies idiosyncratically among ponds for most markers (F).


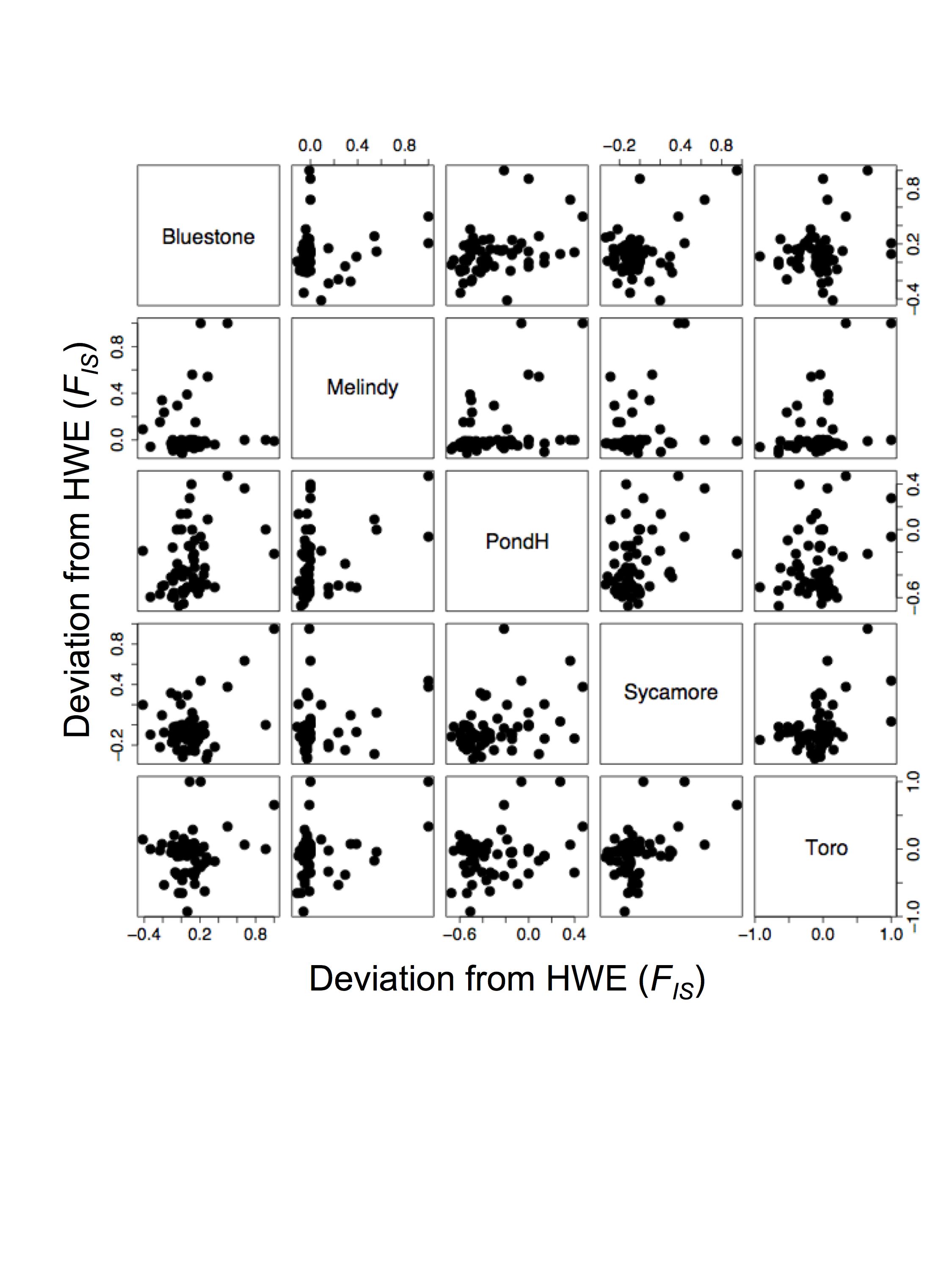


Figure S6 – Scatterplot matrix illustrating weak/no correlation of *FIS* (standardized deviation from HWE) between ponds. Each point represents one marker. Kendall’s tau (nonparametric correlation) and P-value for each comparison are given in Table S2.


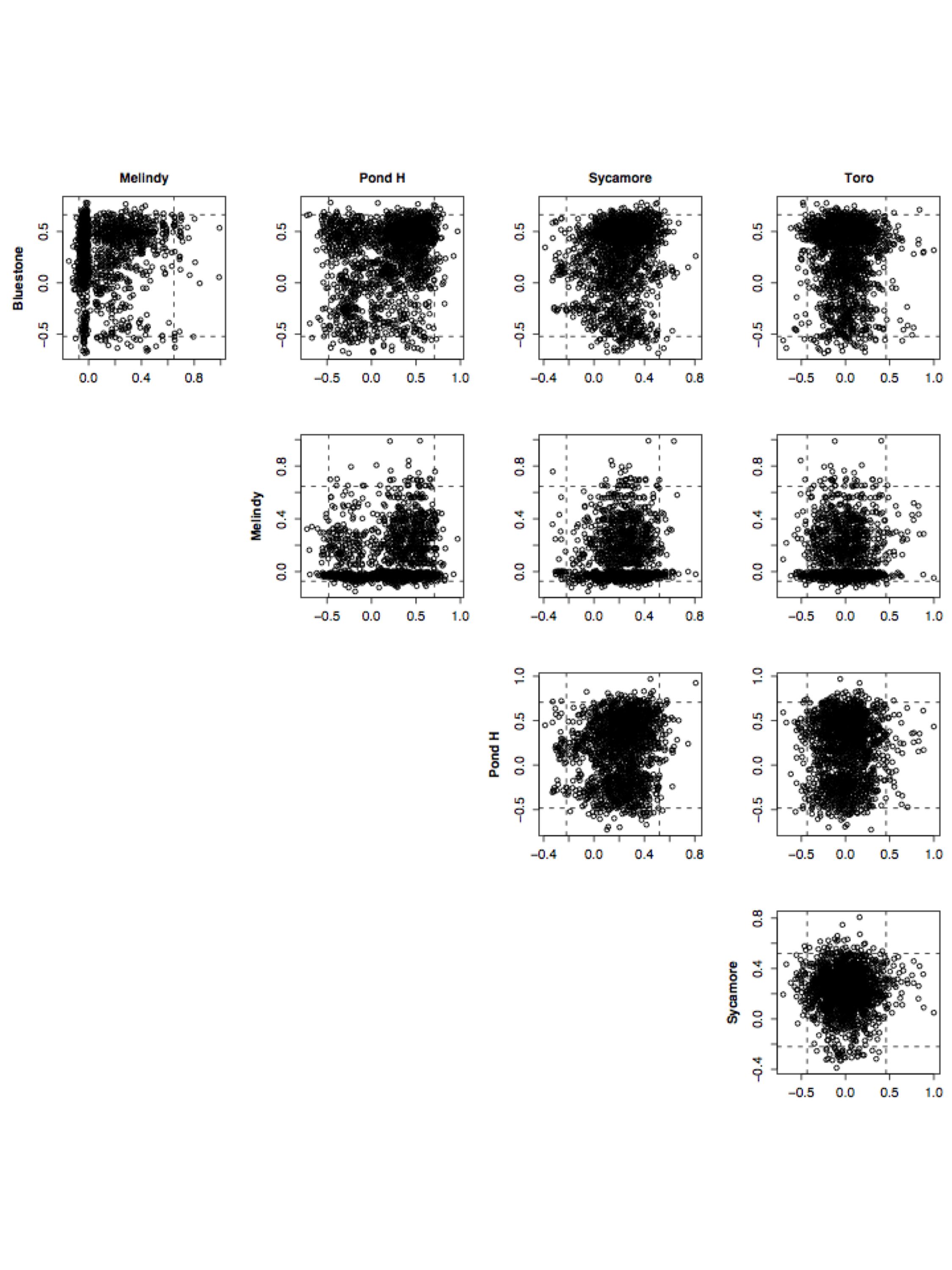


Figure S7 – Scatterplot matrix illustrating weak/no correlation of LD (*r*: the correlation of genotypic indicators) between ponds. Each point represents one pair of markers. Mantel test results are given in Table S3. Dashed lines illustrate the 2.5% and 97.5% quantiles for the distribution of *r* in each pond. Only a few markers are above or below this 95% quantile envelope in more than one pond and no markers are above or below it in all ponds.


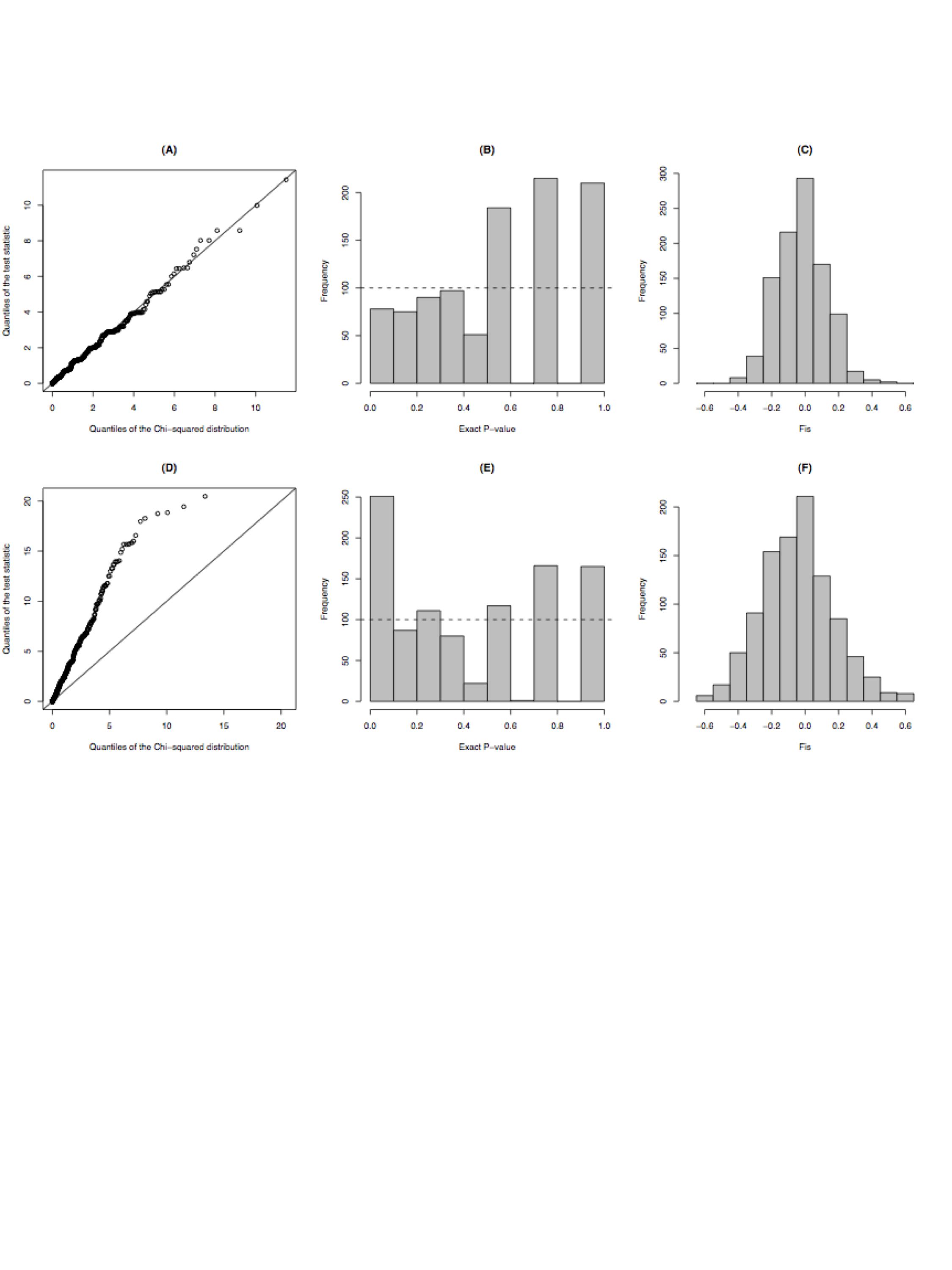


Figure S8 - Tests of goodness-of-fit to Hardy-Weinberg expections when the assumption of random union of gametes is correct (top row of panels) and when 10 random mating pairs produce 500 offspring each (bottom row of panels). (A) and (D) show the quantiles of the observed vs. expected chi-squared distribution for 1000 simulated data sets under each demographic scenario. (B) and (E) show the distribution of Fisher’s exact P-value, and (C) and (F) show the distributions of *FIS*.

| a) |  |  |  |  |  |  |
| --- | --- | --- | --- | --- | --- | --- |
|  | *A*1*A*1 | *A*1*A*2 | *A*2*A*2 |  | *M* |  |
| *B*1*B*1 |  |  |  |  |  | “Simple” asymmetrical Dobzansky- |
| *B*1*B*2 |  |  |  |  |  | Muller incompatibility (DMI) causing |
| *B*2*B*2 |  |  |  |  |  | directional selection |
|  |  |  |  |  |  |  |
| b) |  |  |  |  |  |  |
|  | *A*1*A*1 | *A*1*A*2 | *A*2*A*2 |  |  |  |
| *B*1*B*1 |  |  |  |  |  | True heterozygote disadvantage |
| *B*1*B*2 |  |  |  |  |  |  |
| *B*2*B*2 |  |  |  |  |  |  |
|  |  |  |  |  |  |  |
| c) |  |  |  |  |  |  |
|  | *A*1*A*1 | *A*1*A*2 | *A*2*A*2 |  |  |  |
| *B*1*B*1 |  |  |  |  |  | Symmetrical DMI causing marginal |
| *B*1*B*2 |  |  |  |  |  | heterozygote disadvantage |
| *B*2*B*2 |  |  |  |  |  |  |
|  |  |  |  |  |  |  |
| d) |  |  |  |  |  |  |
|  | *A*1*A*1 | *A*1*A*2 | *A*2*A*2 |  |  |  |
| *B*1*B*1 |  | X | X |  |  | DMI between linked genes causing |
| *B*1*B*2 | X |  | X |  |  | marginal heterozygote disadvantage |
| *B*2*B*2 | X | X |  |  |  |  |
|  |  |  |  |  |  |  |
| e) |  |  |  |  |  |  |
|  | *A*1*A*1 | *A*1*A*2 | *A*2*A*2 |  |  |  |
| *B*1*B*1 |  |  |  |  |  | Additive and epistatic effects causing |
| *B*1*B*2 |  |  |  |  |  | marginal heterozygote advantage |
| *B*2*B*2 |  |  |  |  |  |  |

Figure S9 - Simple genetic models of hybrid dysfunction can result in any pattern of apparent single-locus selection at a linked marker. Pure species genotypes for loci *A* and *B* are *A*1*A*1­*­B*1*­B*1 for species 1 and *A*­2*A*­2­*B*2*B*2 for species 2. Cells in each 3x3 table represent average fitnesses of two-locus genotypes, with darker shading indicating lower fitness. Cells under *M* represent single-locus average fitnesses for a marker linked to locus *B*. The “simple” Dobzhansky-Muller model (a) causes directional selection on *M*. Simple heterozygote disadvantage (b) can appear similar to a symmetrical Dobzhansky-Muller incompatibility (c) or to an asymmetrical incompatibility between linked loci (d) where the crossed out genotypes are rare or absent, and therefore do not affect the marginal fitnesses of the marker *M* genotypes. Even marginal heterozygote advantage can result from certain configurations. In (e), *B*2 tends to have an additive advantage over *B*1 except for a large-effect, recessive incompatibility with *A*1. Finally, because the marginal fitness of an *M* genotype is weighted by genotype frequencies at the interacting loci, the strength and direction of selection are frequency-dependent under many scenarios.
