# Supplementary material for: Rapid fixation of non-native alleles revealed by genome-wide SNP analysis of hybrid tiger salamanders
Source: BMC Evol Biol. 2009 Jul 24;9:176. doi: 10.1186/1471-2148-9-176 (PMC2724412; doi:10.1186/1471-2148-9-176)
Supplement: Additional file 3 — Supplementary Tables S2, S3, and S4. Tables showing cross-pond correlation of deviations from Hardy-Weinberg and linkage disequilibria. [file 1471-2148-9-176-S3.doc]

**Supplementary tables for**

**Fitzpatrick et al. "Rapid fixation of introduced alleles revealed by genome-wide SNP analysis of hybrid tiger salamanders**"

Table S2. Tests of the correlation of *F*IS­ among ponds. Kendall's Tau is above the diagonal and the *P*-value is below the diagonal. The Bonferroni adjusted critical *P-*value for 10 tests is 0.005.

|  | Bluestone | Melindy | Pond H | Sycamore | Toro |
| --- | --- | --- | --- | --- | --- |
| Bluestone |  | 0.0983 | 0.1978 | -0.0195 | -0.0129 |
| Melindy | 0.2637 |  | 0.2443 | 0.1331 | 0.1547 |
| Pond H | 0.0224 | 0.0054 |  | 0.1822 | 0.0010 |
| Sycamore | 0.8216 | 0.1296 | 0.0352 |  | 0.1640 |
| Toro | 0.8821 | 0.0785 | 0.9905 | 0.0584 |  |

Table S3. Tests of the correlation of LD among ponds. The matrix correlation is above the diagonal and the Mantel *P-*value (10,000 randomizations) is below the diagonal. The Bonferroni adjusted critical *P-*value for 10 tests is 0.005.

|  | Bluestone | Melindy | Pond H | Sycamore | Toro |
| --- | --- | --- | --- | --- | --- |
| Bluestone |  | 0.091 | 0.075 | 0.054 | 0.020 |
| Melindy | 0.022 |  | 0.134 | 0.039 | 0.048 |
| Pond H | 0.055 | 0.012 |  | 0.078 | -0.028 |
| Sycamore | 0.118 | 0.411 | 0.052 |  | -0.003 |
| Toro | 0.322 | 0.162 | 0.377 | 0.892 |  |

Table S4. Specimen numbers (HBS collection at University of California, Davis) and collection data for native California Tiger Salamander and introduced Barred Tiger Salamander "controls" used to establish ancestry-informative markers.

| HBS | Locality | Date | Taxon |
| --- | --- | --- | --- |
| 11215 | Fresno (Alta) | 17-Apr-1991 | *Ambystoma californiense* |
| 16917 | Madera (Urrutia) | 15-Apr-1991 | *Ambystoma californiense* |
| 6720 | Alameda (Frick) | 23-May-1986 | *Ambystoma californiense* |
| 11400 | Contra Costa (Diablo) | 27-Apr-1991 | *Ambystoma californiense* |
| 11377 | Alameda (Tesla) | 27-Apr-1991 | *Ambystoma californiense* |
| 14463 | Contra Costa (Vasco) | 15-May-1992 | *Ambystoma californiense* |
| 28366 | Santa Barbara (Black) | 13-May-2000 | *Ambystoma californiense* |
| 109714 | Solano (Olcott) | 1-Apr-2006 | *Ambystoma californiense* |
| 8840 | Solano (Olcott) | 17-Apr-1989 | *Ambystoma californiense* |
| 8842 | Solano (Olcott) | 17-Apr-1989 | *Ambystoma californiense* |
| 8845 | Solano (Olcott) | 17-Apr-1989 | *Ambystoma californiense* |
| 8847 | Solano (Olcott) | 17-Apr-1989 | *Ambystoma californiense* |
| 8848 | Solano (Olcott) | 17-Apr-1989 | *Ambystoma californiense* |
| 8851 | Solano (Olcott) | 17-Apr-1989 | *Ambystoma californiense* |
| 6684 | Solano (Olcott) | 23-May-1986 | *Ambystoma californiense* |
| 6685 | Solano (Olcott) | 23-May-1986 | *Ambystoma californiense* |
| 6686 | Solano (Olcott) | 23-May-1986 | *Ambystoma californiense* |
| 6687 | Solano (Olcott) | 23-May-1986 | *Ambystoma californiense* |
| 14342 | Sonoma (Ludwig) | 6-May-1992 | *Ambystoma californiense* |
| 11667 | Stanislas (Hickman) | 10-May-1991 | *Ambystoma californiense* |
| 26702 | Lake (Fivestar) | 24-Apr-1999 | *Ambystoma tigrinum mavortium* |
| 26703 | Lake (Fivestar) | 24-Apr-1999 | *Ambystoma tigrinum mavortium* |
| 26706 | Lake (Fivestar) | 24-Apr-1999 | *Ambystoma tigrinum mavortium* |
| 26708 | Lake (Fivestar) | 24-Apr-1999 | *Ambystoma tigrinum mavortium* |
| 30942 | Lake (Fivestar) | 29-Feb-2001 | *Ambystoma tigrinum mavortium* |
| 30943 | Lake (Fivestar) | 29-Feb-2001 | *Ambystoma tigrinum mavortium* |
| 30945 | Lake (Fivestar) | 29-Feb-2001 | *Ambystoma tigrinum mavortium* |
| 30947 | Lake (Fivestar) | 29-Feb-2001 | *Ambystoma tigrinum mavortium* |
